# Effects of coiling embolism on blood hemodynamic of the MCA aneurysm: a numerical study

**DOI:** 10.1038/s41598-022-26208-9

**Published:** 2022-12-20

**Authors:** Peiman Valipour

**Affiliations:** Department of Textile Engineering, Clothing and Fashion, Qaemshahr Branch, Islamic Azad University, Qaemshahr, Iran

**Keywords:** Biomedical engineering, Mechanical engineering

## Abstract

One of common endovascular technique for treatment of MCA aneurysm is using coiling gel for limiting of blood stream. In this work, computational fluid dynamic is used for the simulation of the blood hemodynamic inside MCA in existence of coiling gel. This work has tried to visualize the impacts of blood characteristics i.e. hematocrit as a protein related factor on efficiency of coiling fiber inside the aneurysm. Tufts of polyester fibers may be attached to the coil to support thrombosis and platelet aggregation. Blood rheology analysis is done by solving RANS equations and it is assumed that blood stream is non-Newtonian with fluid–solid interaction. OSI and WSS are compared on sac surface area for different stages of blood cycle. Achieved results confirm that the coiling gel substantially decreases the blood circulation inside the aneurysm sac. It is also found that the influence of blood hematocrit decreases when the MCA aneurysm is filled by the coiling gel.

## Introduction

One of the main source for the brain hemorrhage is the rupture of the cerebral aneurysm. Although this may cause fatal diseases on human, the exact source of aneurysm formation is not presented yet^1,2^. The formation and growth of the cerebral aneurysm is the mainly related to the blood rheology and characteristics. The early reports have shown that the blood flow features of Wall shear stress (WSS) and OSI have great impact on the rupture and bleeding of the cerebral aneurysms^3,4^.

The advance in the computational techniques has enabled scientists to calculate this hemodynamics on the wall of the aneurysm^5,6^. Besides, technique of the magnetic resonance angiography (MRA) and computed tomography angiography (CTA) offer valuable information on the shape and feature of the real three-dimensional aneurysm which is essential for the computational modeling. In fact, analysis of the real geometry offers more reliable information about the aneurysm growth and rupture^7–9^.

Although presented results by different scholars are not consistent, there are some identical outcomes and analysis that define the main potential risks related to aneurysm rupture^10,11^. Too overcome these inconsistence, several indices and factors have presented and investigated in recent years^12,13^. The medical phenotype and evolution of aneurysms are not the same, and heterogeneity is noticed. Previous studies showed that transparent, small, type I aneurysms evolved swiftly, while a long time is required for the expansion of type II, thick-walled aneurysms^14–16^. Moreover, more inflammatory permeation in the pathological samples of type II aneurysms are available than those of type I aneurysms.

Aneurysm rupture and growth have two independent haemodynamic corridors: in first one, a positive WSS gradient and a high WSS prompt the cell-mediated formation of a small, thin-walled, translucent aneurysm; in another way, high OSI and a low WSS prompt the expression of inflammatory elements that mediate the formation of a thick-walled, atherosclerotic aneurysm^17,18^. Nevertheless, large-scale clinical experimental evidence did not support this hypothesis. Comprehensive literature survey on estimating rupture risk, growth, and endovascular device assessment indicates that WSS and OSI are the main hemodynamic factors have great impacts^19,20^.

**Figure 1:**
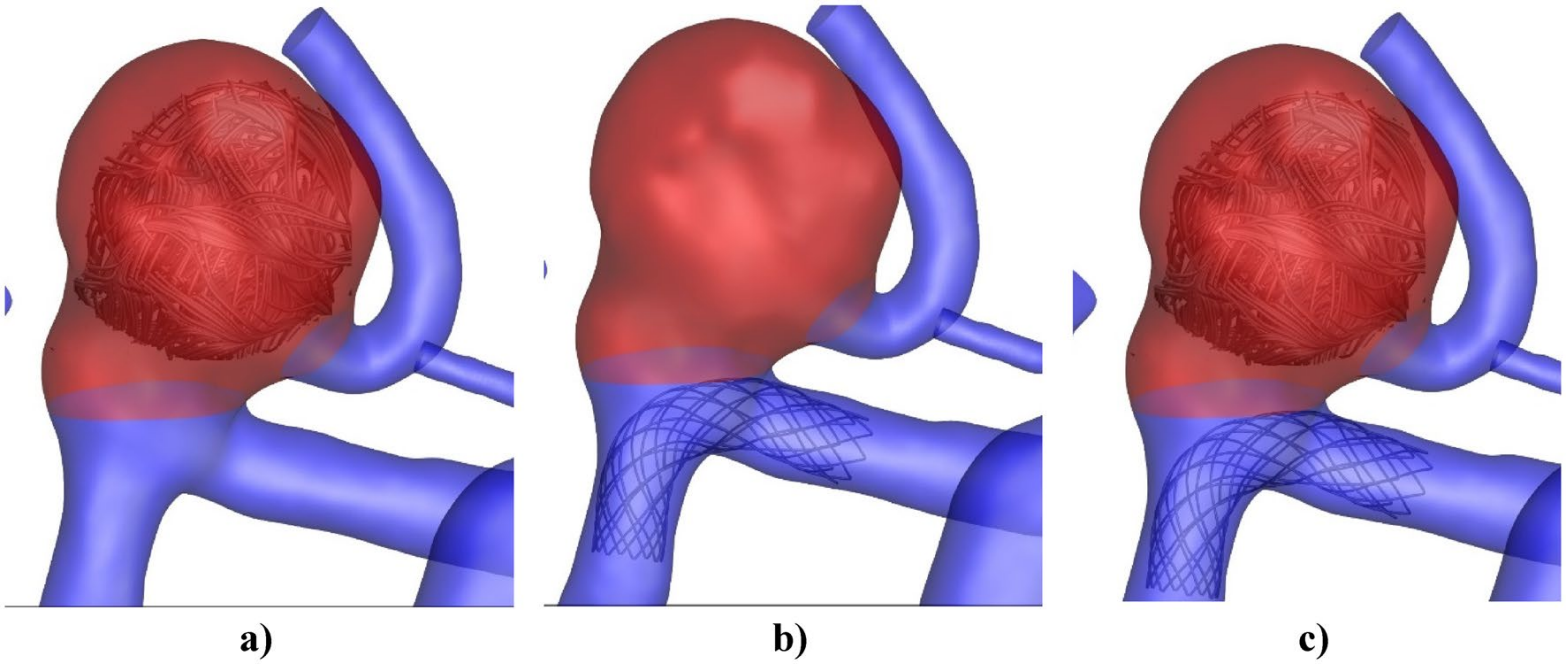
MCA aneurysm with (**a**) coiling (**b**) fiber-based stents stent (**c**) coiling and stent.

One of the most common endovascular technique for treatment of the cerebral saccular aneurysm is coiling technique in which the main portion of the sac are filled with gel (Fig. 1). Shape memory polymers (SMPs) are materials with unlimited potential for this application, because of their versatile and tunable shape memory properties that can be tailored to a patient’s aneurysm geometry and flow condition. Simple coiling denotes to the transfer and packing of detachable coils within the aneurysmal sac via a microcatheter into the aneurysmal dome. The existence of the coil would dense packing and induce rapid blood accumulation formation within the aneurysmal sac, and consequently, sac is separated from the main flow stream. This technique is useful for most of IAs with dome-to-neck ratios (> 2.0). Although several computational studies have done for analysis of blood hemodynamic within cerebral aneurysms, evaluation of the hemodynamic effects of the coiling technique inside MCA aneurysm was not fully investigated in existence articles^21–25^. In this work, blood rheology effects inside the MCA aneurysm with/without coiling are fully investigated.

Drug Eluting Stent (DES) is the Second Generation of Vascular Stent. To solve the problem of In-Stent Restenosis, the bare metal stent as the structural basis is recommended by the researchers and coated it with the biocompatibility coating and anti-proliferative drugs lastly advanced the second-group stent—drug-eluting stent (DES), which means non-absorbable or bioabsorbable polymers or polymer-free stents. The features of stents are altered because of adding polymers, e.g., biomechanics and biocompatibility.

In the present research, computational technique of CFD is applied for the modeling of non-Newtonian, transient blood flow inside real MCA aneurysm. WSS and OSI factors are compared and evaluated in different stages of blood cycle. Influence of blood hematocrit is also investigated on hemodynamic factors.

## Problem description and computational methods

It is confirming that all methods were carried out in accordance with relevant guidelines and regulations. Besides, all experimental protocols were approved by of the Ca' Granda Niguarda Hospital and it is confirmed that informed consent was obtained from all subjects and/or their legal guardian(s).

**Figure 2:**
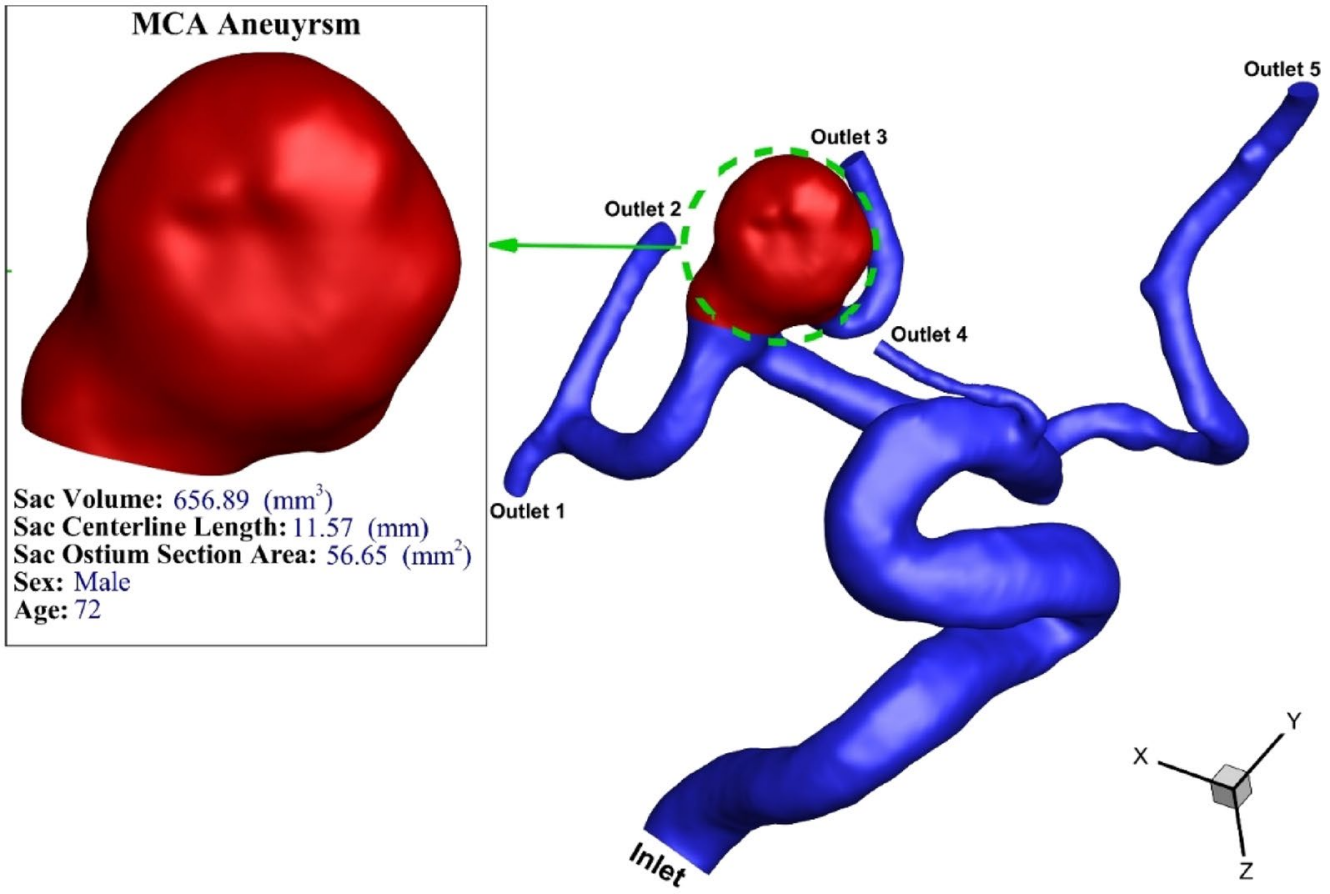
Geometry of chosen MCA aneurysm.

The selected MCA aneurysm is demonstrated in Fig. 2. The geometry of this aneurysm is obtained from Aneurisk webpage^26^. The main reason for selection of this aneurysm is its dome-to-neck ratios which is more than 2.0 and coiling technique is used for the treatment of this aneurysm.

**Figure 3:**
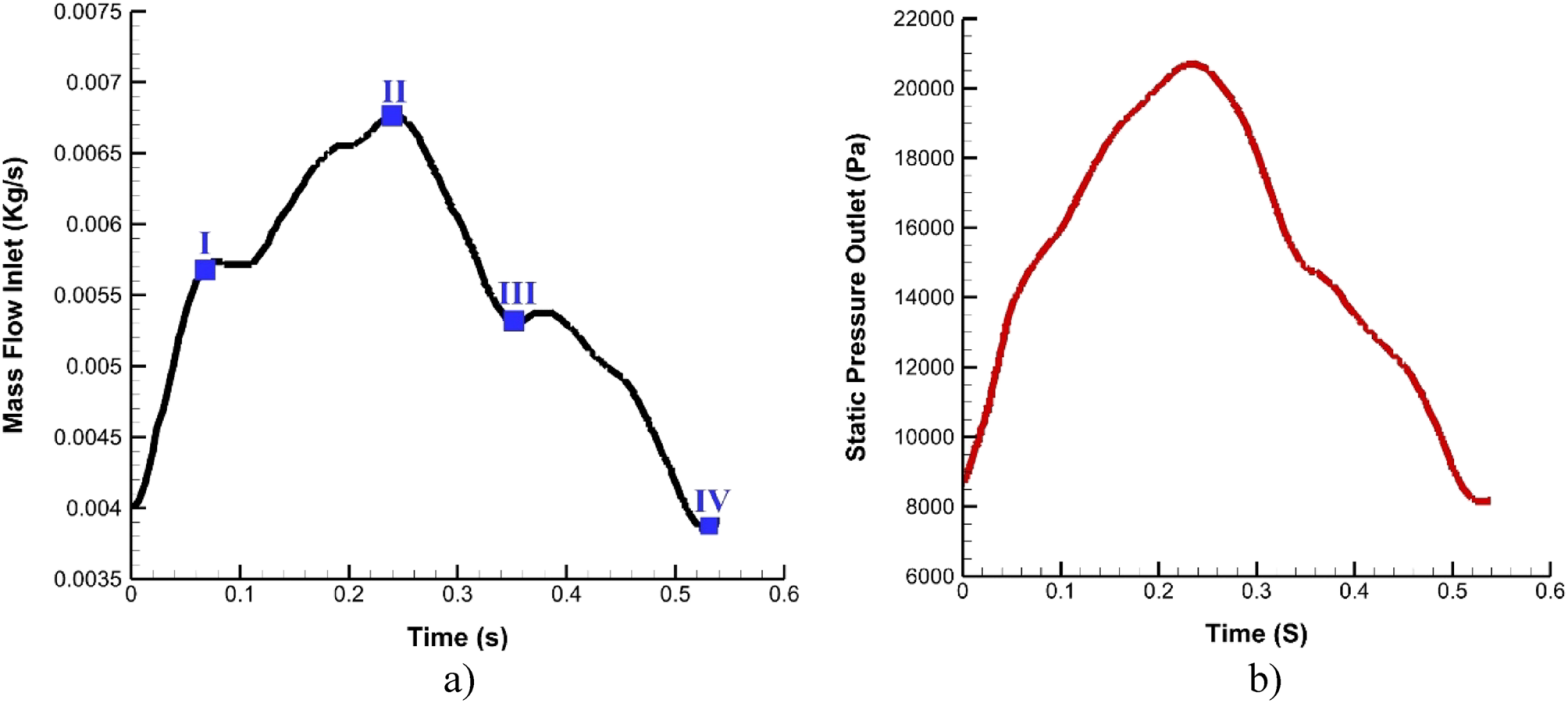
Applied boundary condition at inlets and outlets.

The simulation of bloodstream is done by using simple algorithm with one-way FSI model^27–30^. In this technique, the force from blood stream is applied as exterior force on the structure of the vessel wall^31–33^. Blood stream is assumed non-Newtonian, viscoelastic and transient. The applied boundary condition for inlet is mass flow rate while outlet pressure is applied at outlet of the domain. Figure 3a and 3b demonstrate the applied mass flow rate at inlet and pressure at outlet of domain, respectively. The time step of our simulations is 2 ms. The range of hematocrit is 0.4 to 0.5 in which male hematocrit range is 0.4–0.53. For the estimation of the blood viscosity, Casson model is used since this model calculates the viscosity based on the hematocrit value^34^.

For modeling of coiling, the whole domain inside the sac section is assumed porous media and effects of different coiling fractions are applied via changing the permeability of the porous region as presented in Table 1. In this technique, the porous media is estimated as a layer of solid material with straight parallel pipes of a permanent cross-sectional figure intersecting sample^35^.

**Table 1:** Details of applied coiling for selected MCA aneurysm.

Porosity	L Coil(m)	d Coil (m)	s Coil (m2)	v-Aneurysm (m3)	Inter sur area (1/m)	Prem (m2)	1/Prem
0.75	0.3	2.5 E − 04	2.39E − 04	7.06E − 07	3.38E + 02	1.83E − 06	5.44E + 05
0.85	0.3	2.5 E − 04	2.39E − 04	7.06E − 07	3.38E + 02	2.67E − 06	3.74E + 05

**Figure 4:**
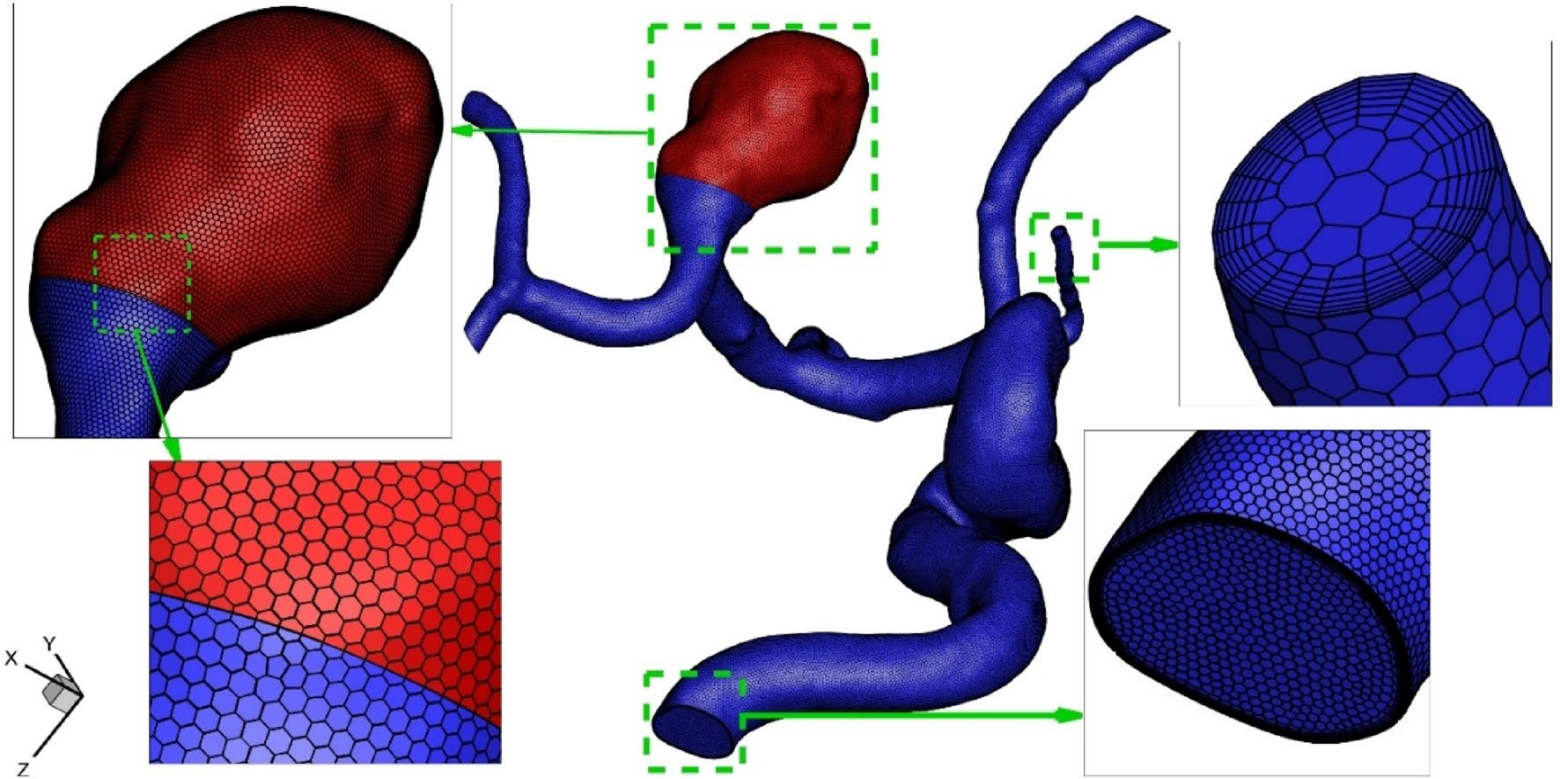
Applied grid for our model.

The produced grid is demonstrated in Fig. 4. The resolution of the generated grid near the aneurysm and vessel wall is higher than other sections since the calculation of the hemodynamic factor should be done with higher precision. Grid study is also performed to find possible relationship between obtained results and size of produced grids. Table 2 present more details about generated grid and results of average WSS for produced grids are compared. The presented results are related to model at peak systolic stage (m = 6.8 mg/s) with HCT = 0.4. It is found that model 3rd with 919,953 cells (with element size of 0.2) is a good option for our investigations.

**Table 2:** Details of used grids.

	Element-size	Number of elements	Ave WSS on Sac (Pa)	Change %
Coarse	0.30	448,379	2	–
Medium	0.25	654,791	2.8	40
Fine	0.20	919,953	3.52	25
Very fine	0.15	1,356,897	3.58	2

## Results and discussion

**Figure 5:**
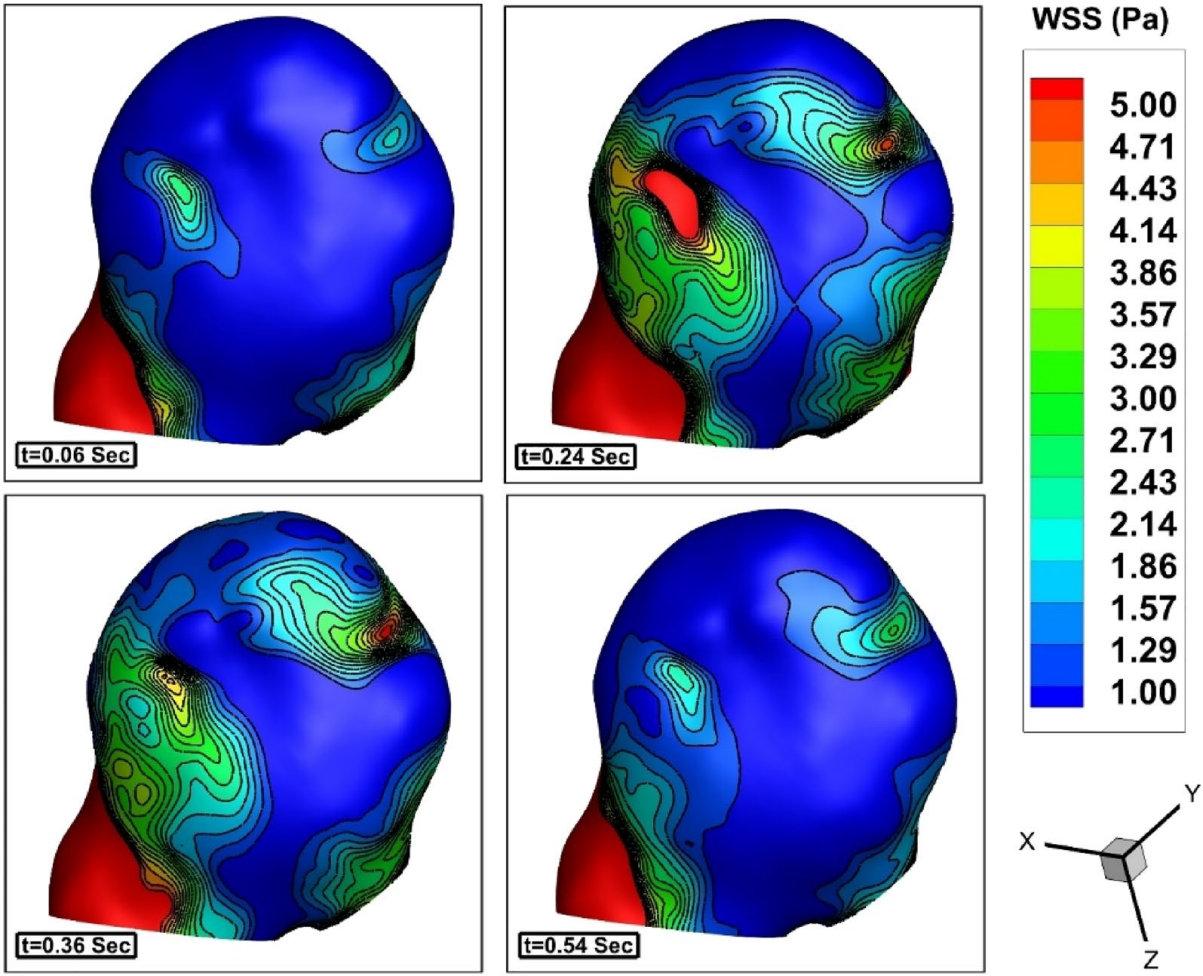
Variation of the WSS on the sac wall in different stages of blood cycle.

**Figure 6:**
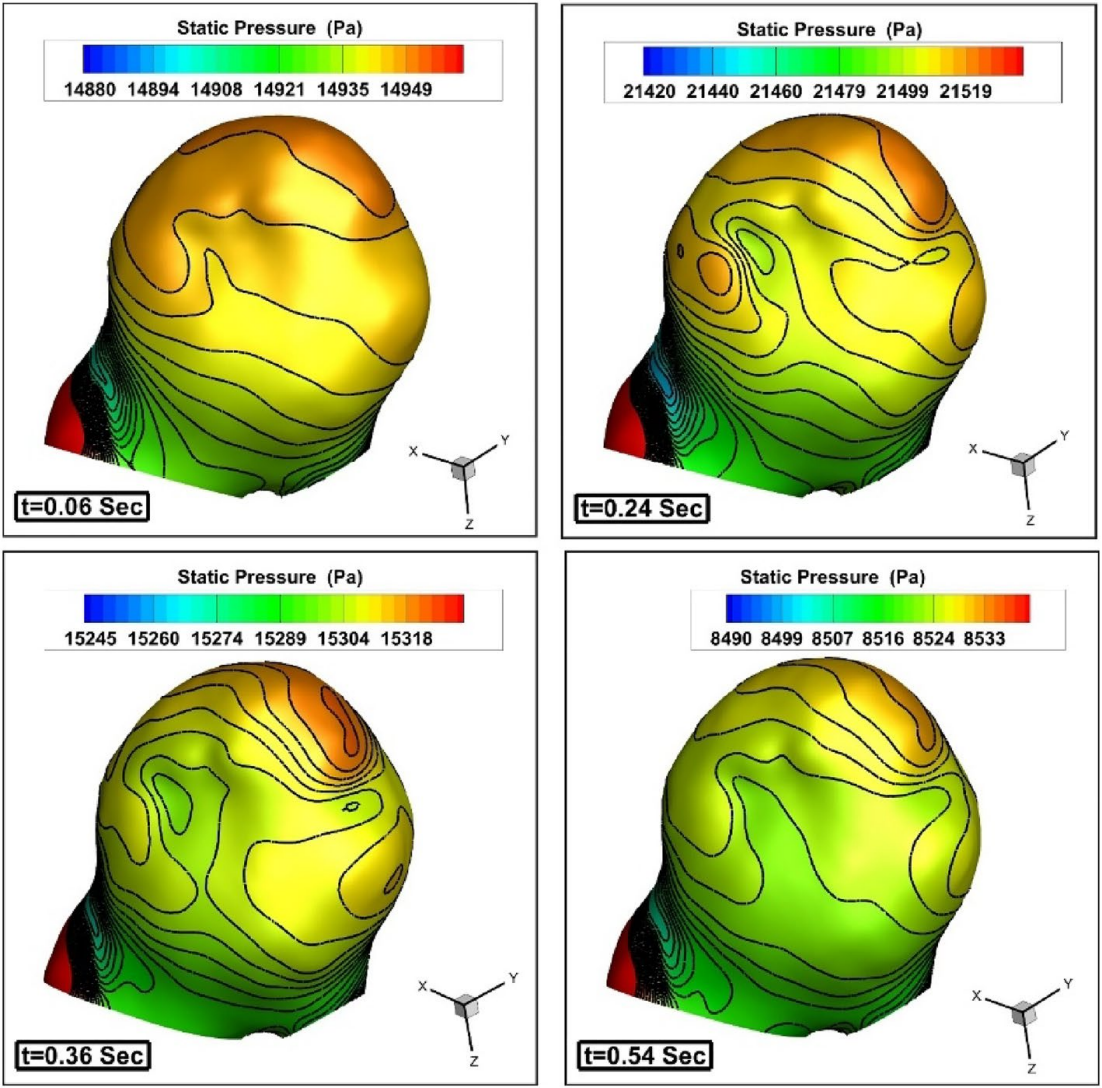
Pressure contour on the sac surface (without coiling).

**Figure 7:**
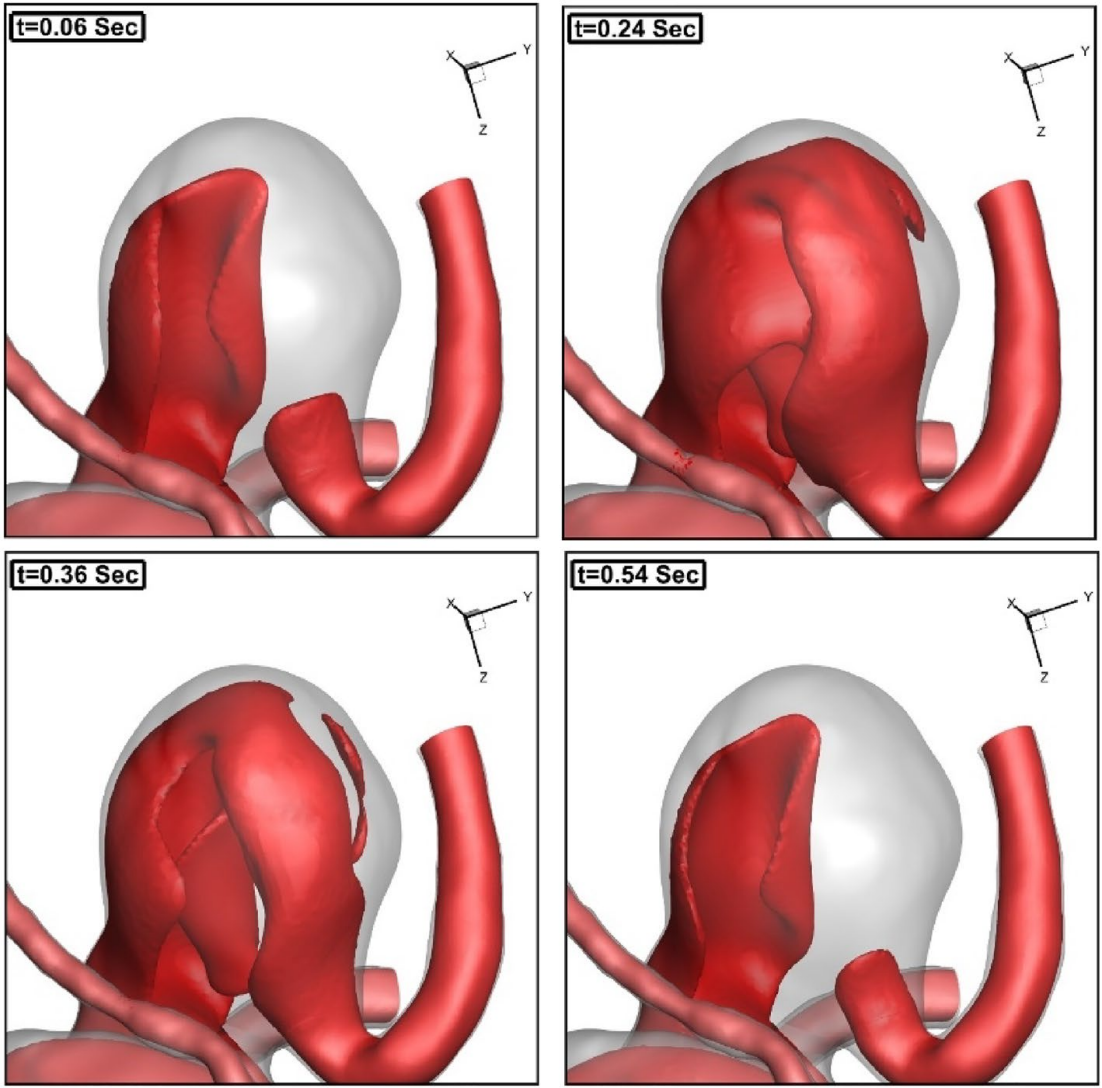
Comparison of is-velocity surface in different stages.

The variation of the WSS on the sac wall in different stages of blood cycle are demonstrated in Fig. 5. In these contours, the porosity of coiling is not applied. As expected, maximum value of WSS initiated at peak systolic stage (t = 0.24 s) and remains in the maximum deceleration (t = 0.36) stage. The contour of pressure on the sac surface is illustrated in Fig. 6 and maximum pressure is noticed in the dome of the aneurysm. Comparison of the blood iso-surface in different stage clearly illustrates the blood hemodynamic in various stages of blood cycle (Fig. 7).

**Figure 8:**
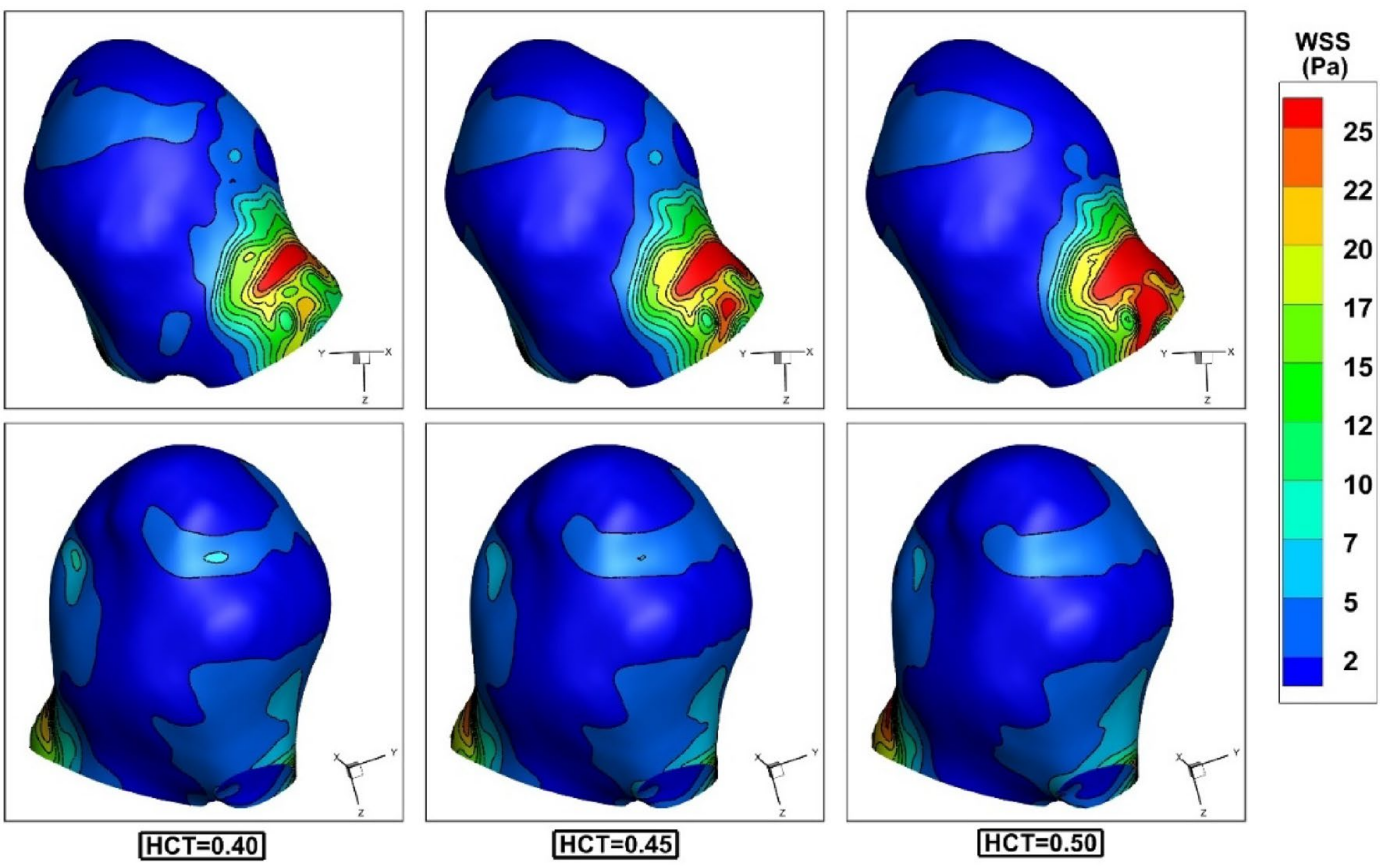
Effects of hematocrit on the WSS distribution at peak systolic.

The influence of the hematocrit on WSS is demonstrated in Fig. 8 at peak systolic stage (t = 0.24 s). The contour shows that increasing blood HCT would increases the WSS near ostium section while WSS near the dome remains unchanged. This is mainly because of the high velocity of the blood stream near the ostium section.

**Figure 9:**
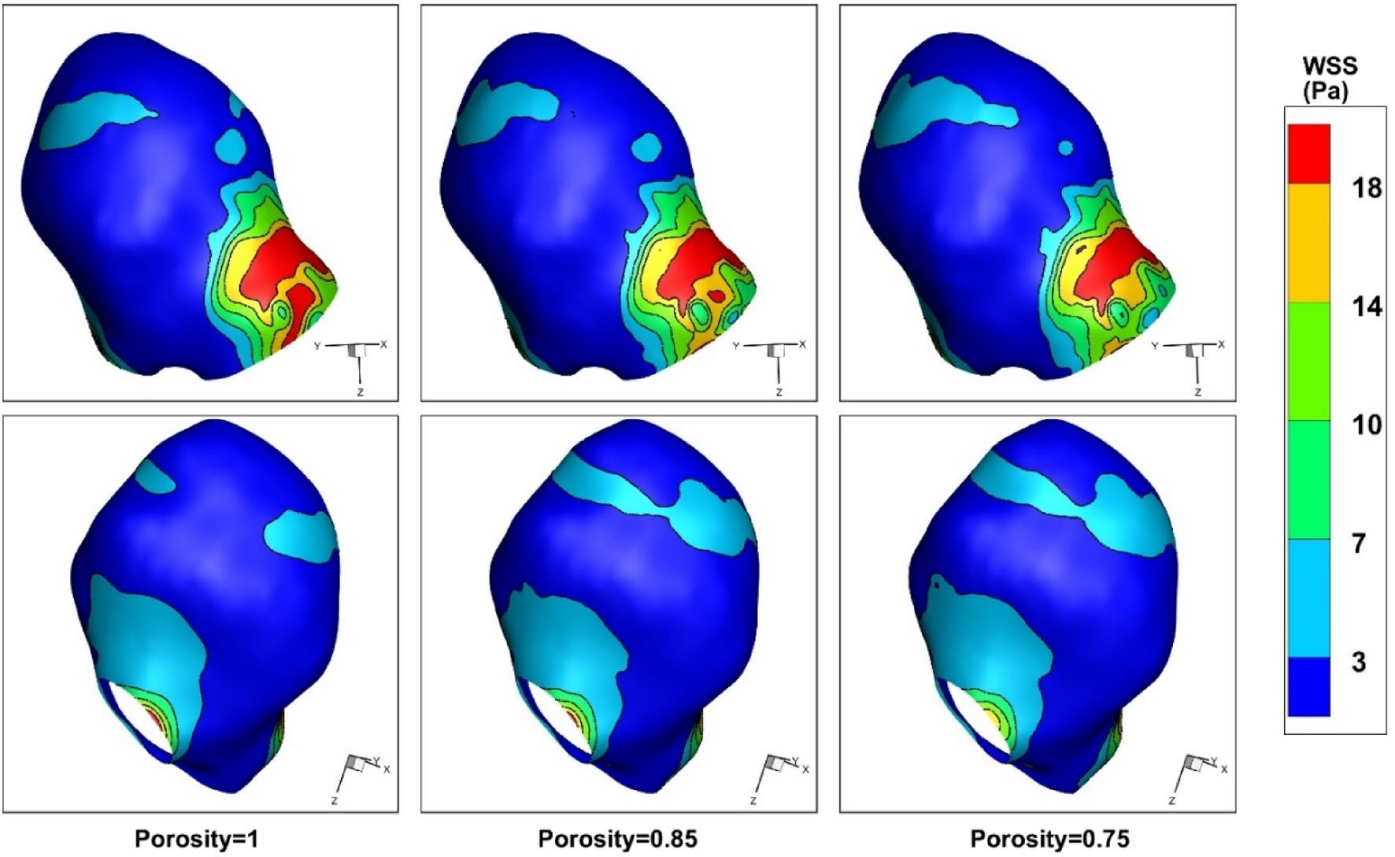
Effects of coiling porosity on WSS at peak systolic.

**Figure 10:**
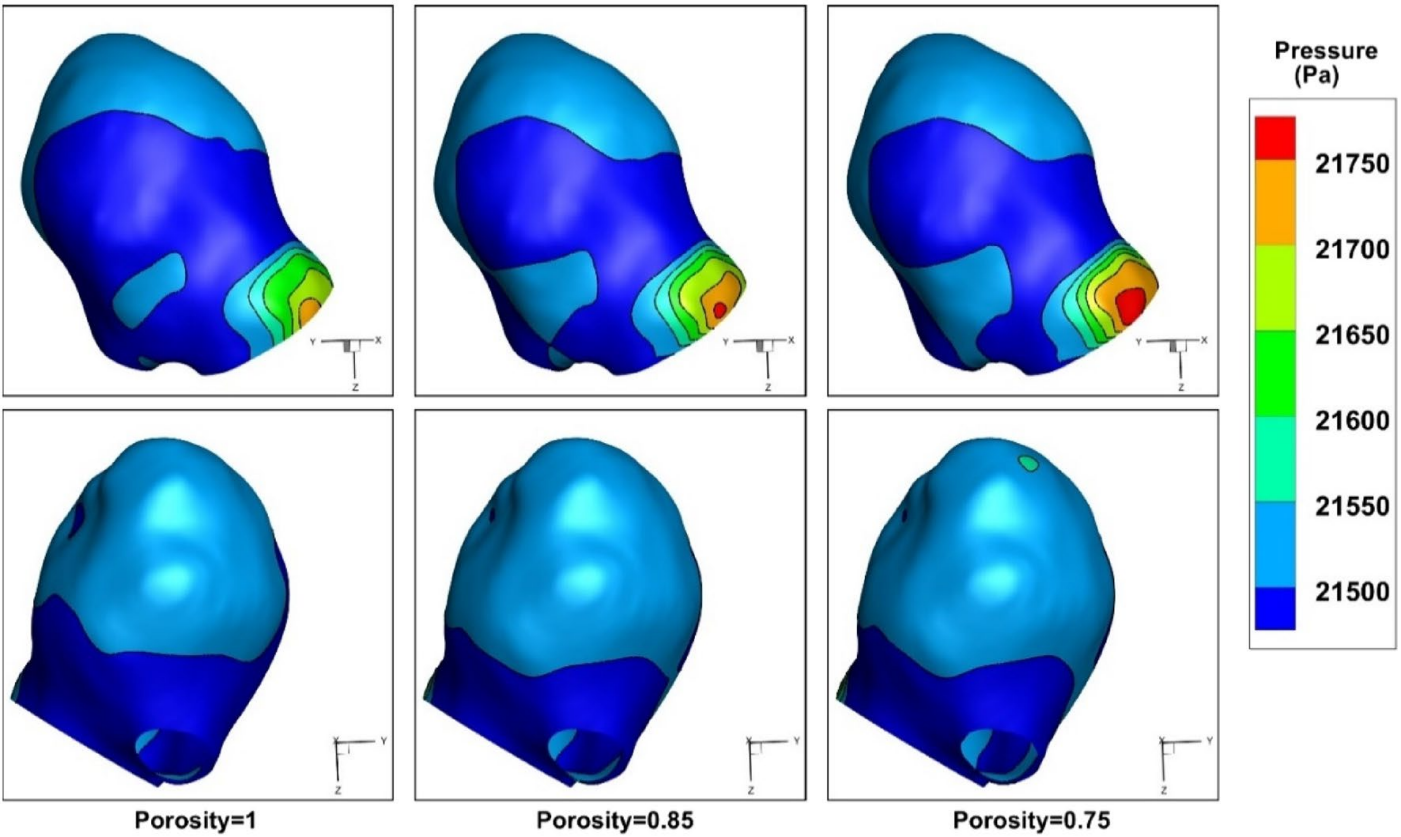
Effects of coiling porosity on pressure distribution at peak systolic.

The influence of the coiling porosity on the distribution of the WSS are demonstrated in Fig. 9. Due to importance of this factor, the variations of this factor are displayed in two sides in this figure. Presented results confirm that the critical region with high WSS happens near the sac ostium section. The variations of the shear stress also defined that the dome of the sac section is less important in the selected model. Previous research shows that the rupture of the aneurysm is more happens in a positive WSS gradient and a high WSS. Thus, ostium section has potential for the rupture. The results of pressure distribution for different porosity values (Fig. 10) also confirm that pressure gradient near sac ostium is higher than other regions at peak systolic.

**Figure 11:**
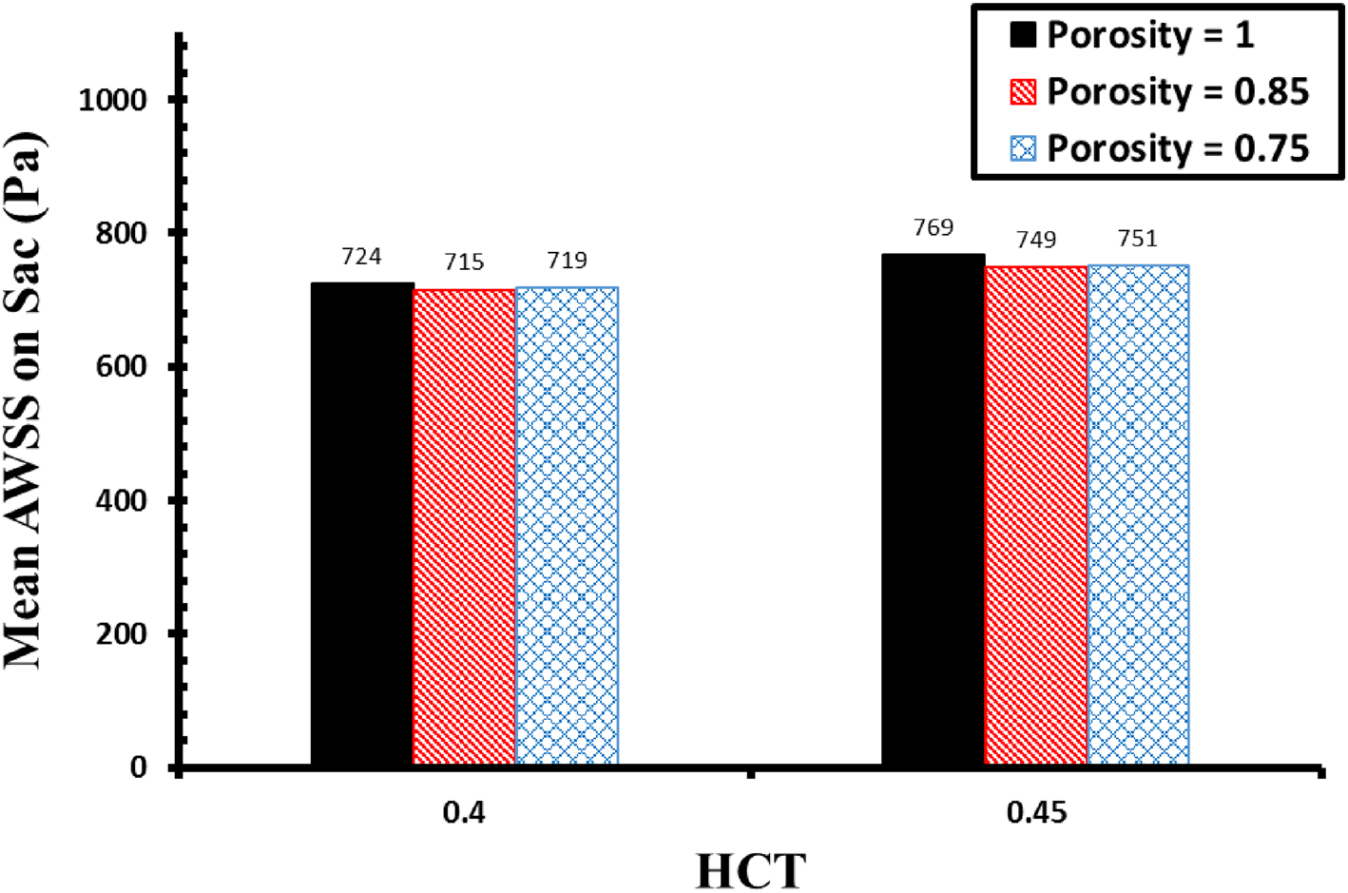
Impacts of HCT and coiling porosity on Mean AWSS on sac surface.

**Figure 12:**
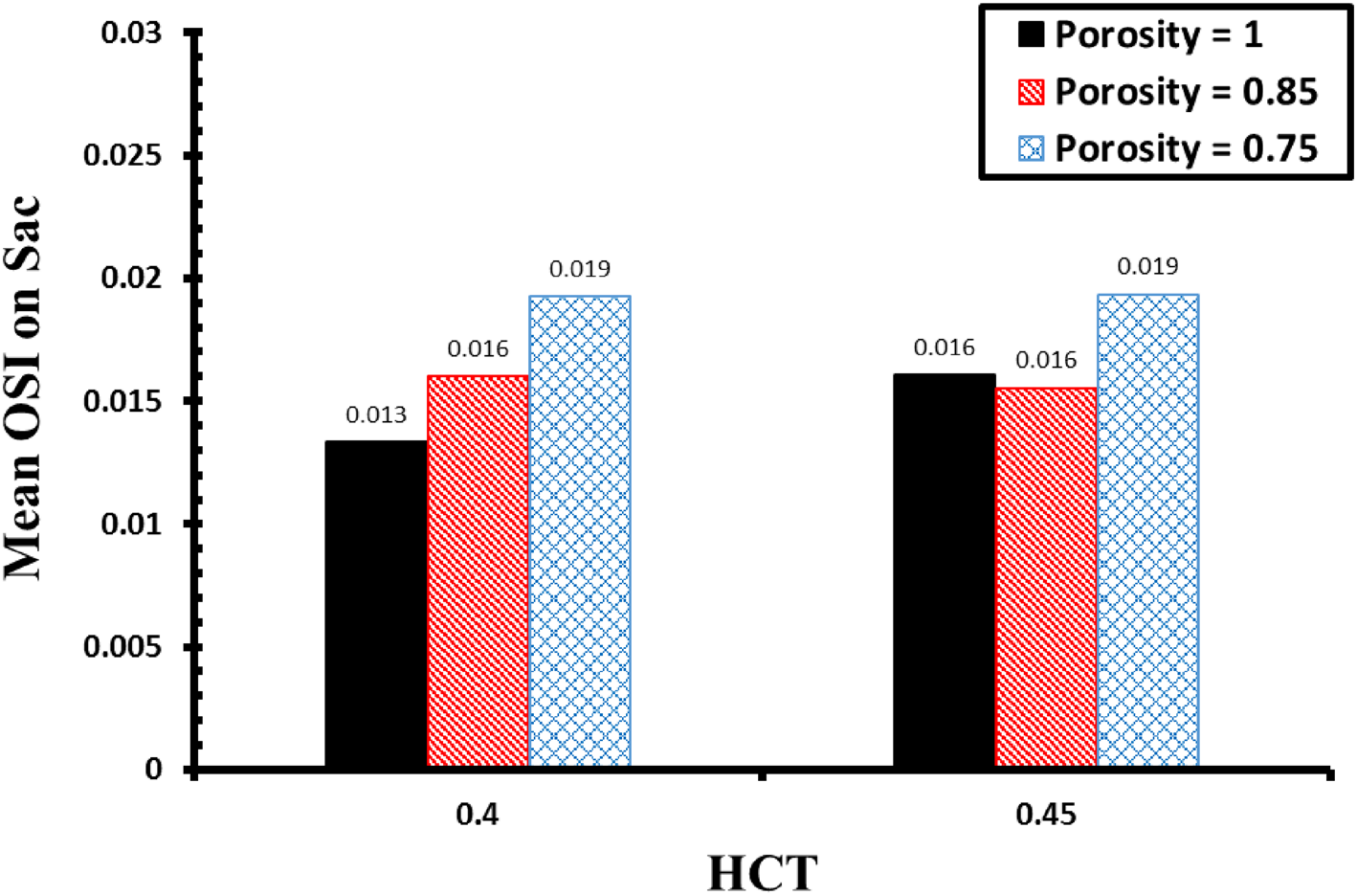
Impacts of HCT and coiling porosity on OSI at end of blood cycle (t = 0.54 s).

Quantitative comparison of the maximum AWSS on sac surface (Fig. 11) demonstrates that increasing the hematocrit rises the maximum WSS on sac surface. Effects of the porosity values has limited impact on this factor. The variation of OSI value calculated at the end of the 3rd cycle is demonstrated in Fig. 12. Achieved results confirm that the effect of the hematocrit (HCT) on the cases with coiling is limited. Meanwhile, decreasing the porosity increases the permeability which means that more domain is filled with coils. Hence, OSI index significantly reduces when aneurysm is filled by coiling gel.

## Conclusion

In present work, the impacts of the coiling on hemodynamic of the blood stream inside MCA aneurysm are investigated. The effect of endovascular coiling is applied by filling the sac section area with porous media. The influences of blood hematocrit value on the distribution of WSS on the sac surface is fully explained. Comparison of the OSI value for different coiling porosities are done to disclose the influence of the coiling technique on the reduction of aneurysm rupture risk. Computational technique of CFD is used for the visualization of the blood flow inside the MCA aneurysm. Our results show that the usage of the coiling considerably reduces the WSS since blood stream circulation is limited in the sac section area. Meanwhile, the blood hematocrit effects are limited by usage of coiling inside the sac region.

## Data Availability

All data generated or analysed during this study are included in this published article.
